# Vision transformer-based stratification of pre/diabetic and pre/hypertensive patients from retinal photographs for 3PM applications

**DOI:** 10.1007/s13167-025-00412-9

**Published:** 2025-05-20

**Authors:** Krithi Pushpanathan, Yang Bai, Xiaofeng Lei, Jocelyn Hui Lin Goh, Can Can Xue, Samantha Min Er Yew, Miaoli Chee, Ten Cheer Quek, Qingsheng Peng, Zhi Da Soh, Marco Chak Yan Yu, Jun Zhou, Yaxing Wang, Jost B. Jonas, Xiaofei Wang, Xueling Sim, E. Shyong Tai, Charumathi Sabanayagam, Rick Siow Mong Goh, Yong Liu, Ching-Yu Cheng, Yih-Chung Tham

**Affiliations:** 1 Department of Ophthalmology, Yong Loo Lin School of Medicine, National University of Singapore, Singapore, Singapore; 2 Centre for Innovation and Precision Eye Health, Yong Loo Lin School of Medicine, National University of Singapore, Singapore, Singapore; 3 Institute of High Performance Computing (IHPC), Agency for Science, Technology and Research (A*STAR), Singapore, Singapore; 4 Singapore Eye Research Institute, Singapore National Eye Centre, Singapore, Singapore; 5 Ophthalmology & Visual Sciences Academic Clinical Programme (EYE ACP), Duke-NUS Medical School, Singapore, Singapore; 6 Ophthalmology and Visual Science Key Lab, Beijing Institute of Ophthalmology, Beijing Tongren Eye Center, Beijing Tongren Hospital, Capital Medical University, BeijingBeijing, China; 7 Rothschild Foundation Hospital, Institut Français de Myopie, Paris, France; 8 Key Laboratory of Biomechanics and Mechanobiology, Ministry of Education, Beijing Advanced Innovation Center for Biomedical Engineering, School of Biological Science and Medical Engineering, Beihang University, Beijing, China; 9 Saw Swee Hock School of Public Health, National University of Singaporeand, National University Health System , Singapore, Singapore; 10 Duke-NUS Medical School, Singapore, Singapore; 11 Precision Health Research, Singapore, Singapore; 12 Department of Medicine, Yong Loo Lin School of Medicine, National University of Singapore, Singapore, Singapore

**Keywords:** Predictive Preventive Personalized Medicine (PPPM / 3PM), Diabetes, Hypertension, Risk stratification, Retinal image, Innovative screening programs, Deep learning, Health risk assessment, Preventable diseases, Protection against health-to-disease transition, Opportunistic screening, Improved individual outcomes

## Abstract

**Objective:**

Diabetes and hypertension pose significant health risks, especially when poorly managed. Retinal evaluation though fundus photography can provide non-invasive assessment of these diseases, yet prior studies focused on disease presence, overlooking control statuses. This study evaluated vision transformer (ViT)-based models for assessing the presence and control statuses of diabetes and hypertension from retinal images.

**Methods:**

ViT-based models with ResNet-50 for patch projection were trained on images from the UK Biobank (*n* = 113,713) and Singapore Epidemiology of Eye Diseases study (*n* = 17,783), and externally validated on the Singapore Prospective Study Programme (*n* = 7,793) and the Beijing Eye Study (*n* = 6064). Model performance was evaluated using the area under the receiver operating characteristic curve (AUROC) for multiple tasks: detecting disease, identifying poorly controlled and well-controlled cases, distinguishing between poorly and well-controlled cases, and detecting pre-diabetes or pre-hypertension.

**Results:**

The models demonstrated strong performance in detecting disease presence, with AUROC values of 0.820 for diabetes and 0.781 for hypertension in internal testing. External validation showed AUROCs ranging from 0.635 to 0.755 for diabetes, and 0.727 to 0.832 for hypertension. For identifying poorly controlled cases, the performance remained high with AUROCs of 0.871 (internal) and 0.655–0.851 (external) for diabetes, and 0.853 (internal) and 0.792–0.915 (external) for hypertension. Detection of well-controlled cases also yielded promising results for diabetes (0.802 [internal]; 0.675–0.838 [external]), and hypertension (0.740 [internal] and 0.675–0.807 [external]). In distinguishing between poorly and well-controlled disease, AUROCs were more modest with 0.630 (internal) and 0.512–0.547 (external) for diabetes, and 0.651 (internal) and 0.639–0.683 (external) for hypertension. For pre-disease detection, the models achieved AUROCs of 0.746 (internal) and 0.523–0.590 (external) for pre-diabetes, and 0.669 (internal) and 0.645–0.679 (external) for pre-hypertension.

**Conclusion:**

ViT-based models show promise in classifying the presence and control statuses of diabetes and hypertension from retinal images. These findings support the potential of retinal imaging as a tool in primary care for opportunistic detection of diabetes and hypertension, risk stratification, and individualised treatment planning. Further validation in diverse clinical settings is warranted to confirm practical utility.

**Supplementary Information:**

The online version contains supplementary material available at 10.1007/s13167-025-00412-9.

## Introduction

Diabetes and arterial hypertension affect 529 million and 1.2 billion people worldwide, respectively, and serve as key modifiable risk factors for cardiovascular disease, the leading cause of death [1, 2]. Despite their prevalence, nearly half of those affected remain undiagnosed, often due to the asymptomatic early stages, leading to delayed intervention and heightened health risks [3–5]. Even among diagnosed individuals receiving treatment, suboptimal disease control is pervasive, exacerbating morbidity and healthcare burdens [2, 6].

Addressing these challenges requires a shift from reactive disease management to a predictive, preventive, and personalised medicine (3PM) paradigm [7–10], where early identification and targeted interventions mitigate disease progression and optimise patient outcomes [11, 12]. The predictive component of 3PM necessitates early risk stratification, ensuring timely identification of undiagnosed, poorly controlled cases, as well as individuals with pre-diabetes and pre-hypertension, before complications arise. The preventive aspect focuses on implementing proactive interventions, reducing the likelihood of progression of diabetes and hypertension. The personalised element involves tailoring management strategies to individual patient profiles, accounting for factors such as comorbidities, genetic predisposition, and lifestyle influences [13, 14]. Effective implementation of 3PM in diabetes and hypertension requires systematic risk stratification tools capable of identifying high-priority cases and optimising resource allocation to improve patient outcomes.

Deep learning techniques applied to retinal photographs offer a promising solution for achieving these objectives [15]. Retinal imaging, which captures changes in the microvascular system, can reveal systemic alterations [16–19] that provide valuable insights into both conditions [17, 20–26]. With increasing integration of retinal imaging into primary care for routine diabetic retinopathy (DR) screening, this non-invasive method could be leveraged to opportunistically assess the presence and control of diabetes and hypertension in a broader patient population.

### Working hypothesis and study aims in the framework of 3PM

Current deep learning models applied to retinal imaging have primarily focused on detecting diabetes and hypertension, but overlook the critical need for disease control stratification in 3PM. While conventional convolutional neural networks (CNNs), such as visual geometry group (VGG) network [17, 24], residual network (ResNet) [20, 21], and inception architectures [22, 25], have proven effective for binary classification tasks, they have not been developed to identify well-controlled and poorly controlled statuses. This gap limits their utility in 3PM, where disease control stratification is essential for targeted referrals, optimised resource allocation, and proactive disease management. Moreover, emerging transformer-based architectures, particularly vision transformers (ViT), offer enhanced capabilities in extracting subtle imaging features, potentially improving predictive accuracy and clinical applicability [27, 28]. However, their use in this domain remains limited; the only transformer-based study focused on hypertension detection without considering control statuses or conducting external validation, restricting clinical relevance [26].

Here, we hypothesise that ViT-based models can effectively detect diabetes and hypertension and stratify their control statuses using retinal photographs. By identifying undiagnosed individuals, those with poorly controlled conditions, and those at risk of progression from pre-diabetes or pre-hypertension, our models can function as assistive tools in clinical decision-making, facilitating timely referrals and personalised management strategies to optimise diabetes and hypertension control and prevent complications. Additionally, we hypothesise that these models can estimate continuous clinical measurements, such as glycated haemoglobin serum concentrations (HbA1c) and blood pressure levels, offering insights into disease control and treatment response [29, 30]. To test these hypotheses, we developed and externally validated these ViT-based models using datasets from large, ethnically diverse cohorts. Our findings assess the generalisability of these models across different clinical contexts and evaluate their potential to enhance predictive accuracy and support clinical decision-making within the 3PM framework.

## Methods

### Study design and population

The development and internal validation of our model were conducted using retinal images from the UK Biobank [31] (UKBB, 113,713 images) and the Singapore Epidemiology of Eye Diseases study [32] (SEED, 17,783 images) datasets (Table 1). These datasets were combined and then randomly allocated into training (70%), validation (15%), and internal test (15%) sets using an individual-specific split such that images for both eyes of an individual were not separated into different subsets. Additionally, external validation was performed on two independent datasets: the Singapore Prospective Study Programme [33] (SP2, 7793 images) and the Beijing Eye Study [34] (BES, 6064 images).

**Table 1:** Characteristics of development and validation datasets

Characteristic	Development and validation sets		External validation sets	
	Singapore Epidemiology of Eye Diseases (SEED)	UK Biobank (UKBB)	Singapore Prospective Study Program (SP2)	Beijing Eye Study (BES)
Country of origin	Singapore	UK	Singapore	China
Camera model	Canon CR-DGi with 10D SLR back	Topcon 3D OCT1000 Mark II	Canon CR-DGi with 10D SLR back	Canon CR6-45 NM
Camera field of view	45°	45°	45°	45°
Image resolution, pixel	Chinese: 3504*2336 Malay: 3072*2048 Indian: 3888*2592	3216*2136	900*600	3888*2592
Total individuals, n	9413	61,598	3999	3146
Total images (n)	17,783	113,713	7793	6064
Age, year, mean (sd)	58.2 (10.1)	56.5 (8.2)	49.8 (11.5)	64.2 (9.6)
Gender, *n* (%)				
*Male*	4648 (49.4)	27,785 (45.1)	1925 (48.1)	1375 (43.7)
*Female*	4765 (50.6)	33,790 (54.9)	2074 (51.9)	1771 (56.3)
Race (*n* (%))	Chinese: 3245 (34.5) Malay: 3090 (32.8) Indian: 3078 (32.7)	White: 55,917 (90.8) Black: 1768 (2.9) Asian: 2024 (3.3) Others: 1448 (2.4)	Chinese: 2363 (59.1) Malay: 862 (21.6) Indian: 771 (19.3) Others: 3 (0.1)	Chinese: 3003 (95.5) Others: 77 (2.4)
Systolic blood pressure, mmHg, mean (sd)	139.3 (21.5)	139.7 (19.6)	132.2 (20.7)	130.7 (20.7)
Diastolic blood pressure, mmHg, mean (sd)	78.4 (10.6)	82.0 (10.7)	78.0 (10.7)	70.2 (12.7)
HbA1c, %, mean (sd)	6.2 (0.8)	5.5 (0.7)	5.6 (0.3)	4.6 (1.5)
Fasting plasma glucose, mmol/L, mean (sd)	NA	NA	5.2 (1.6)	5.7 (1.5)
Random plasma glucose, mmol/L, mean (sd)	6.7 (3.4)	NA	NA	NA
Hypertension classification, *n* (%)				
*Yes*	5895 (62.8)	34,073 (56.0)	1639 (41.4)	1855 (60.0)
*Pre-hypertension*	2204 (23.5)	19,125 (31.4)	1195 (30.2)	568 (18.3)
*Healthy*	1290 (13.7)	7670 (12.6)	1121 (28.3)	674 (21.8)
Hypertension control status*, *n* (%)				
*Poorly controlled*	1824 (30.9)	8497 (24.9)	456 (27.82)	503 (27.1)
*Well-controlled*	1329 (22.5)	3741 (11.0)	208 (12.7)	797 (43.0)
Diabetes classification, *n* (%)				
*Yes*	2707 (28.8)	3318 (5.4)	678 (17.0)	491 (27.7)
*Pre-diabetes*	3850 (41.0)	7718 (12.5)	1165 (29.1)	323 (18.2)
*Healthy*	2855 (30.3)	50,562 (82.1)	2156 (53.9)	957 (54.0)
Diabetes control status*, *n* (%)				
*Poorly controlled*	980 (36.2)	997 (30.0)	172 (25.4)	35 (7.1)
*Well-controlled*	573 (21.2)	1036 (31.2)	52 (7.7)	203 (41.3)

Footnote: *The percentages of poorly or well-controlled diabetes or hypertension are based on individuals with a positive diagnosis who are receiving treatment. As not all diagnosed individuals receive treatment, the total in control categories may not match the overall number of diagnosed cases

All participants provided written informed consent, and all study protocols complied with the principles of the Declaration of Helsinki. Each study received approval from their respective local ethical committees, and we obtained permission from the principal investigator of each study to use the data.

### Image pre-processing

Images from diverse devices and conditions often suffer from poor quality due to factors such as motion blur, incorrect camera settings (e.g. wrong exposure time, fixation setting), and variable illumination [35, 36]. Assessing image quality is crucial for effective model training. To address this, we employed an in-house deep learning algorithm to filter out poor-quality images across all datasets. Additionally, pre-processing included steps such as resizing, normalisation, and data augmentation to optimise the images for model training (Supplementary Material 1). A single macula-centred retinal photograph per eye was used; if multiple images were available, the algorithm identified the highest-quality image for inclusion.

### Definition of diabetes, hypertension, and their control statuses

We defined diabetes, hypertension, and their control statuses according to guidelines from the American Diabetes Association and the American Heart Association, respectively [37, 38] (Supplementary Table 1).

**Fig. 1:**
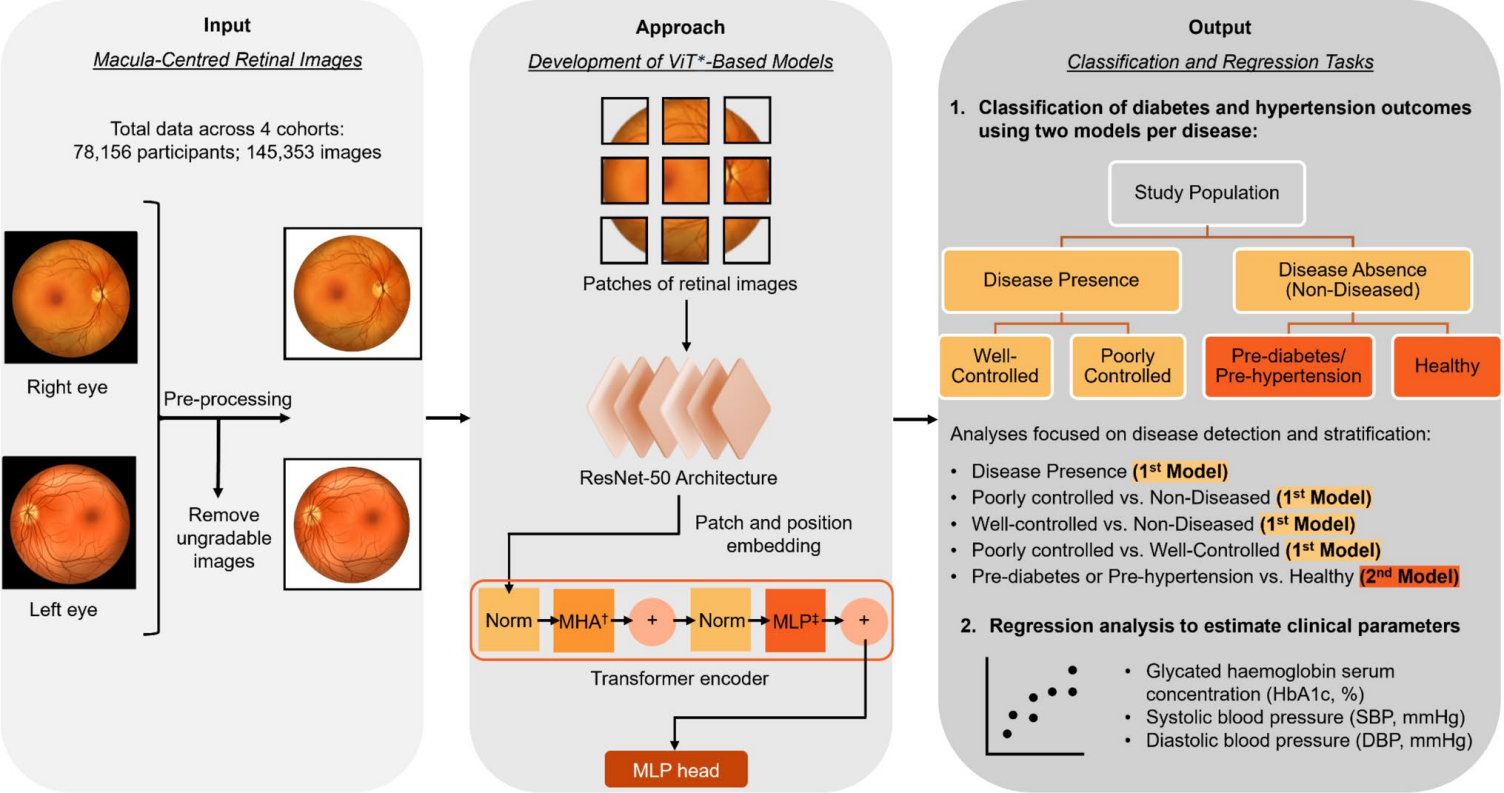
Schematic of the development and evaluation of vision transformer-based models. ViT*, vision transformer; MHA^†^, multi head attention; MLP^‡^, multi-layer perceptron

Pre-diabetes was defined as fasting plasma glucose levels ranging from 5.6 to < 7.0 mmol/L or HbA1c levels between 5.7 and < 6.5%, without self-reported history and treatment. Diabetes was indicated by fasting plasma glucose ≥ 7.0 mmol/L, casual plasma glucose ≥ 11.1 mmol/L, HbA1c ≥ 6.5%, self-reported history of diabetes, use of diabetic medication, or a previous physician diagnosis of diabetes. Diabetic patients who received treatment were further categorised as well-controlled, if the HbA1c was < 7%, or poorly controlled if HbA1c was ≥ 7%. Individuals who did not minimally meet the criteria for pre-diabetes were classified as healthy individuals (Fig. 1).

Pre-hypertension was defined as systolic blood pressure (SBP) ranging from 120 to < 140 mmHg, or diastolic blood pressure (DBP) between 80 and < 90 mmHg, without self-reported history and treatment. Hypertension was indicated by SBP ≥ 140 mmHg, DBP ≥ 90 mmHg, self-reported history of hypertension, or use of anti-hypertensive medication. Hypertensive patients who received treatment were further categorised as well-controlled if both, the SBP was < 140 mmHg and the DBP was < 90 mmHg, or as poorly controlled if either the SBP was ≥ 140 mmHg and/or the DBP was ≥ 90 mmHg. Individuals who did not minimally meet the criteria for pre-hypertension were classified as healthy individuals (Fig. 1).

### Development of the deep learning models

The classification and stratification of diabetes and hypertension control statuses were performed using ViT-based models applied to pre-processed macula-centred retinal images and their corresponding clinical labels. Our methodology closely adheres to the standard ViT base architecture [39], with a key modification involving the substitution of the linear patch projection layer with a ResNet-50-based feature projection. This hybrid architecture combines the strengths of both ViT, which excels at capturing long-range patterns and global image features, and ResNet, which is adept at extracting intricate local features. This integration is designed to potentially enhance the models’ predictive accuracy [40].

Four classification models were designed, each generating probabilities for a distinct outcome: (1) presence of diabetes, (2) presence of pre-diabetes, (3) presence of hypertension, and (4) presence of pre-hypertension (Fig. 1 and Supplementary Tables 2 and 3). The same models used to detect disease presence (models 1 and 3) were also applied to categorise individuals into poorly controlled and well-controlled states, without additional fine-tuning. In these models, the absence of disease includes both pre-diabetic/pre-hypertensive and healthy individuals.

The analysis focused on detecting (1) disease presence, (2) poorly controlled cases (compared to non-diseased individuals), (3) well-controlled cases (compared to non-diseased individuals), (4) differentiating between poorly controlled and well-controlled cases, and (5) identifying pre-diabetes or pre-hypertension (distinguished from healthy individuals). These analyses were conducted separately for diabetes and hypertension across the entire population. Additionally, a sub-analysis was performed to evaluate the same five hypertension outcomes within the subset of individuals with diabetes.

In addition to the classification models, regression models were trained on the retinal images to predict the relevant continuous biomarker values, including HbA1c levels (%), SBP (mmHg), and DBP (mmHg).

Data bootstrapping was applied to improve model performance and address class imbalance by increasing the number of positive samples through resampling, bringing their representation to 150% of the original count. This potentially improved on the model’s ability to learn relevant features and improved its reliability in detecting and classifying disease states.

### Saliency maps

Saliency maps were generated using the Grad-CAM method [41] to highlight the most influential regions within retinal images, aiding in the understanding of features crucial for predictive accuracy regarding diabetes and hypertension control statuses by our model. In these heatmaps, warmer hues such as red and orange indicate regions with higher activation, contributing more significantly to the model’s output. Conversely, cooler hues such as blue and green signify areas of inactivity, suggesting less influence on the model’s decision-making process.

### Evaluation and statistical analysis

We assessed the ViT-based models’ performance in class discrimination using area under the receiving operator curve (AUROC), sensitivity and specificity at the optimal threshold derived from the Youden index, and maximum F1 score. Results were evaluated at the participant level by averaging scores from both eyes. To gauge metric uncertainty, we employed bootstrapping with 2000 iterations, deriving a 95% confidence interval (CI) from the resulting distribution of AUROC values.

For the regression models targeting continuous outcomes, performance evaluation included root mean square error (RMSE), mean absolute error (MAE), standard deviation (SD), and coefficient of determination (R^2^). Bland–Altman plots were generated to assess the agreement between predicted and observed values. To further investigate potential errors, a Student’s *t*-test was conducted to assess systematic bias, identifying any consistent deviations, while a regression analysis of residuals was performed to test for proportional bias, indicating any scaling errors.

Statistical analyses and performance metrics were computed using R (Version 4.3.1, R Foundation, Vienna, Austria). Statistical significance was considered at *p* < 0.05 for all tests.

## Results

The ViT-based models were developed, validated, and externally tested using 145,353 macula-centred retinal images (78,156 individuals) from four diverse Asian and European cohorts (SEED, UKBB, SP2, and BES) (Table 1). No significant differences were found in the demographic and clinical characteristics between the training and internal testing populations, ensuring balanced representation and minimising potential biases (Supplementary Fig. 1).

### Model performance in detecting diabetes and control status

**Fig. 2:**
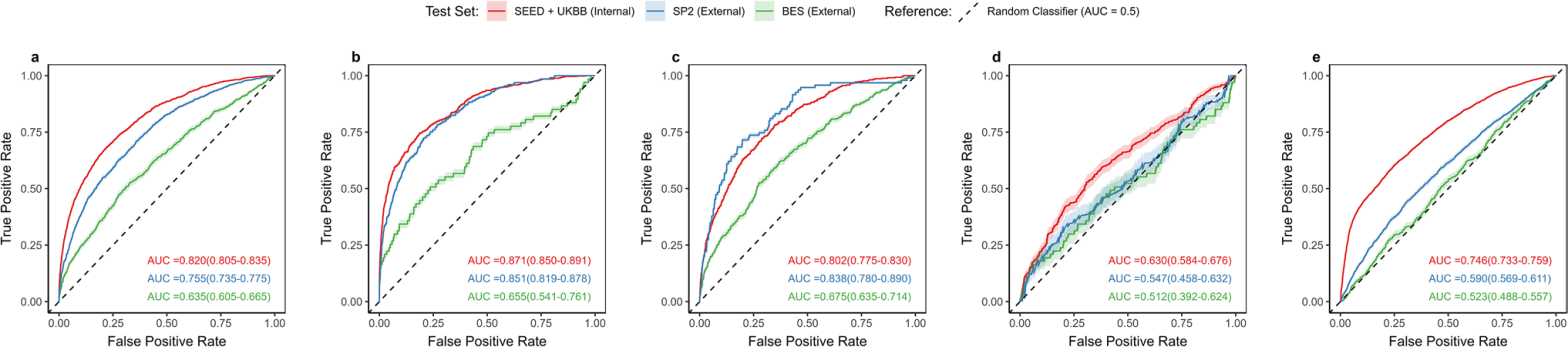
Receiver operating curves demonstrating the classification performance of vision transformer-based models from retinal fundus images across various diabetes outcomes. **a** Presence of diabetes**, b** poorly controlled diabetes (compared to non-diseased individuals),** c** well-controlled diabetes (compared to non-diseased individuals), **d** differentiating poorly controlled and well-controlled diabetes, **e** pre-diabetes (compared to healthy individuals)

Table 2 and Fig. 2 summarise the performance of ViT-based models across diabetes detection and classifying control status.

**Table 2:** Performance metrics of custom vision transformers in classifying diabetes outcomes

*Test set*	*No. of images*	*AUROC*	*95% CI*	*Sensitivity at optimal threshold*	*Specificity at optimal threshold*	*SE when SP = 0.8*	*Max F1 score*
***Presence of diabetes***							
*SEED + UKBB (internal)*	1642:18,105	0.820	0.805–0.835	0.729	0.760	0.678	0.420
*SP2 (external)*	1301:6492	0.755	0.735–0.775	0.773	0.602	0.546	0.444
*BES (external)*	943:2472	0.635	0.605–0.665	0.501	0.716	0.375	0.463
***Poorly controlled diabetes (compared to non-diseased individuals)***							
*SEED + UKBB (internal)*	535:18,105	0.871	0.850–0.891	0.707	0.862	0.753	0.401
*SP2 (external)*	326:6492	0.851	0.819–0.878	0.698	0.844	0.715	0.388
*BES (external)*	67:2472	0.655	0.541–0.761	0.514	0.799	0.486	0.255
***Well-controlled diabetes (compared to non-diseased individuals)***							
*SEED + UKBB (internal)*	432:18,105	0.802	0.775–0.830	0.678	0.779	0.640	0.217
*SP2 (external)*	95:6492	0.838	0.780–0.890	0.904	0.647	0.712	0.173
*BES (external)*	390:2472	0.675	0.635–0.714	0.591	0.691	0.394	0.332
***Poorly controlled diabetes vs. well-controlled diabetes***							
*SEED + UKBB (internal)*	535:432	0.630	0.584–0.676	0.570	0.657	0.400	0.714
*SP2 (external)*	326:95	0.547	0.458–0.632	0.221	0.923	0.326	0.871
*BES (external)*	67:390	0.512	0.392–0.624	0.200	0.926	0.257	0.265
***Pre-diabetes (compared to healthy individuals)***							
*SEED + UKBB (internal)*	3220:14,904	0.746	0.733–0.759	0.685	0.669	0.548	0.459
*SP2 (external)*	2256:4236	0.590	0.569–0.611	0.599	0.548	0.333	0.520
*BES (external*	628:1844	0.523	0.488–0.557	0.204	0.854	0.241	0.406

Footnote: Sensitivity and specificity at the optimal threshold were determined using the Youden index. *SE* sensitivity, *SP* specificity

The model achieved an internal AUROC of 0.820 (95% CI 0.805–0.835) detecting diabetes presence, with external AUROCs of 0.755 (95% CI 0.735–0.775) in SP2 and 0.635 (95% CI 0.605–0.665) in BES (Fig. 2a). For predicting HbA1c levels, however, the regression model demonstrated limited accuracy, with internal R^2^ of 0.264 and external R^2^ values of 0.067 (BES) and 0.168 (SP2) (Supplementary Table 4). The internal dataset exhibited lower RMSE (0.656), MAE (0.386), and SD (0.656) compared to external test sets, where RMSE values were 1.042 (SP2) and 2.008 (BES), MAE values were 0.670 (SP2) and 1.856 (BES), and SD values were 1.029 (SP2) and 1.142 (BES). Bias analysis revealed significant systematic and proportional bias (*p* < 0.001), corroborated by Bland–Altman plots, exhibiting a diagonal upward trend, indicating scaling errors with larger discrepancies at higher predicted values (Supplementary Fig. 2).

The AUROC values for detecting poorly controlled diabetes (compared to non-diabetic individuals) were 0.871 (0.850–0.891) internally, 0.851 (0.819–0.878) for SP2, and 0.655 (0.541–0.761) for BES (Fig. 2b). Sensitivity at a specificity threshold of 0.8 was also highest for this classification, with values of 0.753 internally and 0.715 (SP2) and 0.486 (BES) externally.

For detecting well-controlled diabetes (compared to non-diabetic individuals), the internal AUROC was 0.802 (0.775–0.830), with external AUROCs of 0.838 (0.780–0.890, SP2) and 0.675 (0.635–0.714, BES) (Fig. 2c).

Within the diabetic cohort, model performance for differentiating poorly controlled from well-controlled diabetes achieved an internal AUROC of 0.630 (0.584–0.676), with external AUROCs of 0.547 (0.458–0.632, SP2) and 0.512 (0.392–0.624, BES) (Fig. 2d). Sensitivity at a specificity threshold of 0.8 was lowest for this class, with values of 0.400 internally and 0.326 (SP2) and 0.257 (BES) externally (Table 2).

For detecting pre-diabetes (compared to healthy individuals), the internal AUROC was 0.746 (0.733–0.759), with external AUROCs of 0.590 (0.569–0.611, SP2) and 0.523 (0.488–0.557, BES) (Fig. 2e).

Across all diabetes-related classifications, performance was consistently poorest in the BES dataset, with a relative AUROC reduction of 16–30% compared to the internal test set. Additionally, while F1 scores remained low for most classifications ($\le$ 0.520), the highest scores were observed when distinguishing poorly controlled from well-controlled diabetes in the internal (0.714) and SP2 (0.871) datasets—the only cases where the positive class (i.e., poorly controlled) outnumbered the negative class (i.e., well-controlled).

### Model performance in detecting hypertension and control status

**Fig. 3:**
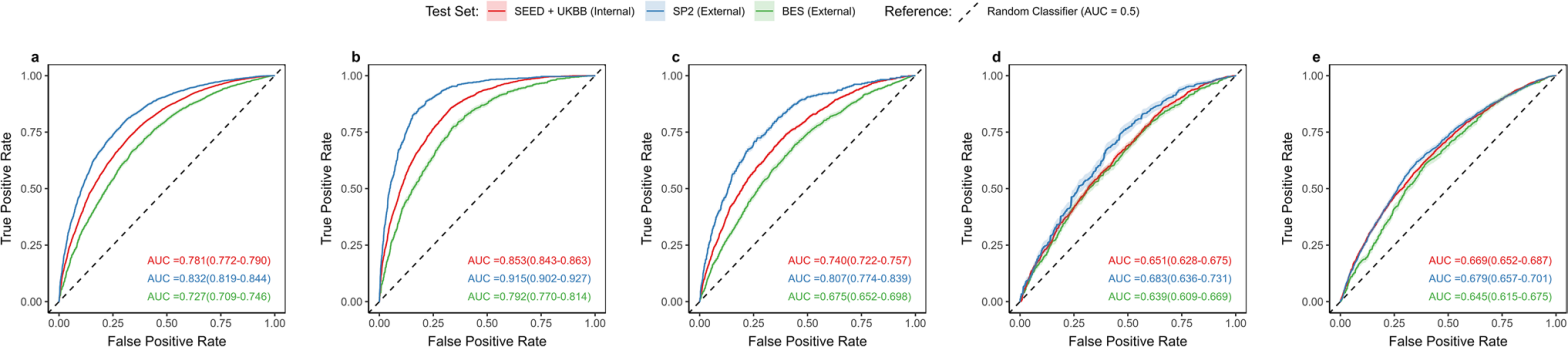
Receiver operating curves demonstrating the classification performance of vision transformer-based models from retinal fundus images across various hypertension outcomes. **a** Presence of hypertension, **b** poorly controlled hypertension (compared to non-diseased individuals), **c** well-controlled hypertension (compared to non-diseased individuals), **d** differentiating poorly controlled and well-controlled hypertension, **e** pre-hypertension (compared to healthy individuals)

Table 3 and Fig. 3 summarise the performance of ViT-based models in detecting hypertension status and classifying control status.

**Table 3:** Performance metrics of custom vision transformers in classifying hypertension outcomes

*Test set*	*No. of images*	*AUROC*	*95% CI*	*Sensitivity at optimal threshold*	*Specificity at optimal threshold*	*SE when SP* = *0.8*	*Max F1 score*
***Presence of Hypertension***							
*SEED* + *UKBB (internal)*	11,081:8483	0.781	0.772–0.790	0.738	0.690	0.593	0.775
*SP2 (external)*	3081:4551	0.832	0.819–0.844	0.778	0.743	0.703	0.726
*BES (external)*	3547:2422	0.727	0.709–0.746	0.774	0.575	0.487	0.774
***Poorly controlled hypertension (compared to non-diseased individuals)***							
*SEED* + *UKBB (internal)*	2847:8483	0.853	0.843–0.863	0.849	0.708	0.727	0.634
*SP2 (external)*	827:4551	0.915	0.902–0.927	0.883	0.814	0.889	0.650
*BES (external)*	964:2422	0.792	0.770–0.814	0.706	0.738	0.600	0.597
***Well-controlled hypertension (compared to non-diseased individuals)***							
*SEED* + *UKBB (internal)*	1433:8483	0.740	0.722–0.757	0.737	0.639	0.520	0.399
*SP2 (external)*	341:4551	0.807	0.774–0.839	0.744	0.748	0.653	0.342
*BES (external)*	1526:2422	0.675	0.652–0.698	0.715	0.565	0.409	0.602
***Poorly controlled hypertension vs. well-controlled hypertension***							
*SEED* + *UKBB (internal)*	2847:1433	0.651	0.628–0.675	0.694	0.525	0.363	0.808
*SP2 (external)*	827:341	0.683	0.636–0.731	0.756	0.551	0.368	0.846
*BES (external)*	964:1526	0.639	0.609–0.669	0.690	0.514	0.338	0.584
***Pre-hypertension (compared to healthy individuals)***							
*SEED* + *UKBB (internal)*	5954:2519	0.669	0.652–0.687	0.575	0.682	0.407	0.830
*SP2 (external)*	2335:2216	0.679	0.658–0.700	0.677	0.598	0.412	0.701
*BES (external)*	1107:1315	0.645	0.614–0.677	0.701	0.525	0.349	0.649

Footnote: Sensitivity and specificity at the optimal threshold were determined using the Youden index. *SE* sensitivity, *SP* specificity

For detecting hypertension presence, the model achieved an internal AUROC of 0.781 (0.772–0.790) and external AUROCs of 0.832 (0.819–0.844, SP2) and 0.727 (0.709–0.746, BES) (Fig. 3a). In contrast, regression models showed limited accuracy in predicting SBP and DBP values, with R^2^ values of 0.191 (internal), 0.294 (SP2), and 0.071 (BES) for SBP, and 0.086 (internal), 0.126 (SP2), and 0.019 (BES) for DBP (Supplementary Table 4). Performance was generally superior in the internal test set, with lower RMSE, MAE, and SD, and higher R^2^, compared to the external test sets. However, an exception was observed in the prediction of SBP, where SP2 demonstrated higher RMSE (19.587 vs. 17.855) and MAE (16.074 vs. 14.119), but achieved a higher R^2^ (0.294 vs. 0.191) than the internal test set. Systematic and proportional bias (*p* < 0.001) was observed in SBP and DBP predictions across all datasets, except for DBP predictions in the internal dataset, where no systematic bias was detected (*p* = 0.162) (Supplementary Table 4).

The AUROC values for detecting poorly controlled hypertension (compared to non-hypertensive individuals) were 0.853 (0.843–0.863) internally, 0.915 (0.902–0.927) for SP2, and 0.792 (0.770–0.814) for BES (Fig. 3b). Sensitivity at a specificity threshold of 0.8 was highest for this subgroup, with values of 0.727 for the internal test set, 0.600 for BES, and 0.889 for SP2.

For detecting well-controlled hypertension (compared to non-hypertensive individuals), the model achieved an internal AUROC of 0.740 (0.722–0.757) and external AUROCs of 0.807 (0.774–0.839, SP2) and 0.675 (0.652–0.698, BES) (Fig. 3c).

Within the hypertensive cohort, model performance for differentiating poorly controlled from well-controlled hypertension achieved an internal AUROC of 0.651 (0.628–0.675) and external AUROCs of 0.683 (0.636–0.731, SP2) and 0.639 (0.609–0.669, BES) (Fig. 3d). Sensitivity at a specificity threshold of 0.8 was lowest for this classification, with values of 0.363 internally and 0.368 (SP2) and 0.338 (BES) externally (Table 3).

For detecting pre-hypertension (compared to healthy individuals), the internal AUROC was 0.669 (0.652–0.687) and external AUROCs were 0.679 (0.658–0.700, SP2) and 0.645 (0.614–0.677, BES) (Fig. 3e).

Unlike diabetes-related outcomes, where both external datasets showed larger performance declines compared to the internal dataset, BES exhibited a smaller AUROC reduction (2–9%) for hypertension outcomes, while SP2 outperformed the internal dataset across all classifications. Furthermore, F1 scores were higher across all classifications and datasets for hypertension outcomes than for diabetes outcomes.

### Model performance in detecting hypertension and control status among diabetics

**Fig. 4:**
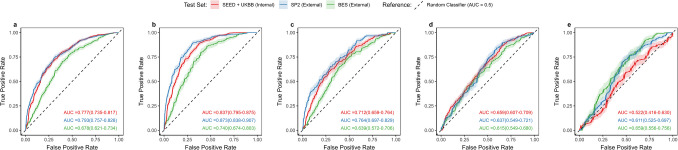
Receiver Operating Curves Demonstrating the Classification Performance of Vision Transformer-Based Models from Retinal Fundus Images Across Various Hypertension Outcomes among People with Diabetes**. a** Presence of hypertension,** b** poorly controlled hypertension (compared to non-diseased individuals),** c** well-controlled hypertension (compared to non-diseased individuals),** d** differentiating poorly controlled and well-controlled hypertension, **e** Pre-hypertension (compared to healthy individuals)

Hypertension outcomes among diabetics revealed relatively poorer model performance compared to the general population (Table 4 and Fig. 4).

**Table 4:** Performance metrics of custom vision transformers in classifying hypertension outcomes among diabetic patients

*Test set*	*No. of images*	*AUROC*	*95% CI*	*Sensitivity at optimal threshold*	*Specificity at optimal threshold*	*SE when SP* = *0.8*	*Max F1 score*
***Presence of hypertension***							
*SEED* + *UKBB (internal)*	1304:326	0.777	0.735–0.817	0.710	0.759	0.595	0.904
*SP2 (external)*	873:394	0.793	0.757–0.828	0.683	0.772	0.616	0.847
*BES (external)*	681:229	0.678	0.621–0.734	0.728	0.603	0.388	0.864
***Poorly controlled hypertension (compared to non-diseased individuals)***							
*SEED* + *UKBB (internal)*	577:326	0.837	0.795–0.875	0.808	0.759	0.700	0.859
*SP2 (external)*	322:394	0.873	0.838–0.907	0.844	0.772	0.775	0.798
*BES (external)*	209:229	0.740	0.674–0.803	0.927	0.496	0.486	0.743
***Well-controlled hypertension (compared to non-diseased individuals)***							
*SEED* + *UKBB (internal)*	347:326	0.712	0.659–0.764	0.639	0.741	0.476	0.724
*SP2 (external)*	119:394	0.764	0.697–0.829	0.667	0.728	0.540	0.525
*BES (external)*	326:229	0.639	0.572–0.706	0.696	0.603	0.327	0.751
***Poorly controlled hypertension vs. well-controlled hypertension***							
*SEED* + *UKBB (internal)*	577:347	0.659	0.607–0.709	0.690	0.545	0.367	0.786
*SP2 (external)*	322:119	0.637	0.549–0.721	0.850	0.413	0.260	0.854
*BES (external)*	209:326	0.615	0.549–0.680	0.661	0.542	0.330	0.599
***Pre-hypertension (compared to healthy individuals)***							
*SEED* + *UKBB (internal)*	249:69	0.522	0.416–0.630	0.797	0.333	0.173	0.881
*SP2 (external)*	273:121	0.611	0.525–0.697	0.736	0.500	0.221	0.827
*BES (external)*	135:94	0.659	0.556–0.756	0.903	0.388	0.403	0.779

Footnote: Sensitivity and specificity at the optimal threshold were determined using the Youden index. *SE* sensitivity, *SP* specificity

The strongest AUROC results was observed for detecting poorly controlled hypertension (compared to non-hypertensive individuals), with an internal AUROC of 0.837 (0.795–0.875), and external AUROCs of 0.873 (0.838–0.907, SP2) and 0.740 (0.674–0.803, BES) (Fig. 4b). Sensitivity at a specificity threshold of 0.8 was also strongest for this classification, with values of 0.700 internally, 0.775 for SP2, and 0.486 for BES.

Meanwhile, the weakest AUROC performance was observed for detecting pre-hypertension (compared to healthy individuals), with an internal AUROC of 0.522 (0.416–0.630) and external AUROCs of 0.611 (0.525–0.697, SP2) and 0.659 (0.556–0.756, BES) (Fig. 4e). Sensitivity at a specificity threshold of 0.8 in this classification was 0.1 73 internally, 0.221 for SP2, and 0.403 for BES.

Additionally, F1 scores were generally higher across most classifications and datasets for hypertension outcomes in people with diabetes compared to the general population.

### Saliency maps

The saliency maps indicate that the models predominantly focussed on the retinal vasculature, particularly the major blood vessels, when evaluating images for both diabetes and hypertension (Supplementary Figs. 3, 4 and 5). For hypertension, additional emphasis was placed on the region surrounding the optic disk (Supplementary Figs. 4 and 5).

## Discussion

In this study, we developed and validated ViT-based models to classify the presence and control statuses of diabetes and hypertension, leveraging 145,353 macula-centred retinal photographs from 78,156 participants across four diverse Asian and European cohorts. The models demonstrated promising performance in detecting both conditions and stratifying their control statuses, supporting the hypothesis that ViT-based models applied to retinal imaging can potentially aid diabetes and hypertension management, in line with the 3PM framework. However, the models showed limited ability to predict continuous clinical parameters such as HbA1c and blood pressure. These findings partially support our hypotheses, affirming the potential of ViT-based retinal imaging for classifying diabetes and hypertension and stratifying their control statuses, while emphasising the need for further refinement to enhance its predictive power for continuous biomarkers and its broader clinical applicability.

### Unmet patient needs in reactive medicine

The traditional reactive approach to medicine often fails to address the needs of individuals with undiagnosed or poorly controlled diabetes and hypertension, as interventions typically occur only after onset, often when complications have already developed [8, 9]. Many affected individuals remain unaware of their condition, due to the asymptomatic nature of the early stages of these conditions, exacerbating long-term health risks [3, 4]. This gap in early detection highlights a critical unmet patient need in reactive medicine.

Additionally, therapeutic inertia remains a major barrier in reactive medicine. Many patients with diabetes or hypertension continue with suboptimal treatment regimens due to a lack of timely reassessments or clinical uncertainty, leading to prolonged periods of poor disease control [42, 43]. This inertia contributes to avoidable disease progression and increased risk of complications, jeopardising patient outcomes.

### 3PM innovation to improve individual outcomes

The study highlights the potential for these models to assist clinical decision-making and addressing unmet patient needs in the reactive approach to medicine. Their predictive utility lies in individualised patient profiling [9]—identifying individuals with undiagnosed or poorly controlled conditions, as well as those at an elevated risk of progression, such as those with pre-diabetes and pre-hypertension. By facilitating earlier detection of these individuals, the models ensure timely referrals and targeted interventions, integral to the preventive approach within 3PM. This proactive management can potentially help to curb diabetes and hypertension progression and reduce the risk of complications, ultimately improving patient outcomes.

At the same time, by providing insights to an individual’s control status, the models enable healthcare providers to facilitate personalised treatment alterations. This tailored care ensures that treatment plans are aligned with the patient’s unique needs, ultimately leading to better outcomes and sustained health improvements. Furthermore, recognising well-controlled cases helps optimise resource allocation, preventing over-diagnosis and ensuring that healthcare efforts are directed towards individuals who require urgent care [8].

In primary care settings, where retinal imaging is increasingly available, these models further facilitate the opportunistic detection of diabetes and hypertension, along with their control statuses, addressing a key unmet need in reactive medicine. Unlike the traditional approach, where interventions often occur only after disease onset, often after complications have already developed due to the asymptomatic nature of these conditions in their early stages [8, 9], these models ensure earlier intervention. By integrating these models into routine clinical practice, healthcare delivery can be more efficient, patient management can be streamlined, and outcomes can be significantly improved. This shift represents a substantial move away from reactive approaches, paving the way for more proactive and effective healthcare strategies.

### Comparative performance of proposed ViT-based models against existing literature

Our study explores ViT-based models as a promising approach for retinal image-based detection of diabetes and hypertension, demonstrating competitive performance with established deep learning architectures. For diabetes, our model achieved an internal AUROC of 0.820 (0.805–0.835), outperforming a ResNet-18 model (AUROC: 0.731 [0.707–0.756]) that lacked external validation [20]. However, it fell short compared to a ResNet-50 model, which reported AUROCs ranging from 0.788 to 0.932 across internal and two external test sets [21]. Notably, the latter study was conducted exclusively on Chinese cohorts, potentially inflating performance due to limited generalisability. To our knowledge, no prior studies have reported transformer-based models for diabetes detection.

For hypertension detection, our ViT-based model achieved an internal AUROC of 0.781 (0.772–0.790), outperforming both an Inception-v3 model (AUROC, 0.766) [22] and a transformer-based RETFound model (AUROC, 0.690 [0.657–0.724]) [26]. Neither of these studies conducted external validation, highlighting the added value of our approach in testing across diverse datasets.

Across both disease classifications, the models consistently demonstrated the highest AUROC in detecting poorly controlled cases (compared to non-diseased individuals). This could be attributed to the more pronounced retinal features associated with poorly controlled disease states, which facilitate stronger model discrimination. This stratification is particularly valuable for identifying high-risk patients who require urgent intervention and supports targeted clinical interventions to mitigate disease progression.

In regression analysis for the relevant systemic biomarkers (HbA1c, SBP, and DBP), our models consistently yielded low R^2^ values (Supplementary Table 4). This contrasts with the relatively promising AUROC values observed in some classification tasks for diabetes and hypertension outcomes, suggesting that the models may lack critical predictors or interactions influencing biomarker levels. Moreover, these biomarkers are inherently variable due to factors like acute stress or illness [44], or abrupt dietary changes (e.g. high-sugar or salt intake), further complicating accurate regression modelling. Nonetheless, previous studies support some findings; for instance, HbA1c has been reported with MAEs of 0.33 and 1.39 and corresponding R^2^ values of 0.13 and 0.09 [17, 45], which show some overlap with our observed MAE range of 0.386–1.856 and R^2^ range of 0.067–0.264 across internal and external test sets. However, for SBP, our MAEs (14.119–18.533) and R^2^ values (0.071–0.294) were less favourable than those reported in prior studies (MAEs of 9.29 and 11.35, R^2^ of 0.31 and 0.36). Similarly, for DBP, our MAEs (8.092–14.591) and R^2^ values (0.086–0.126) fell short of the reported benchmarks (MAEs of 6.42 and 7.20, R^2^ of 0.32 and 0.35) [17, 45]. The discrepancies between our study’s findings and those of previous studies may stem from factors such as population differences, imaging protocols, feature extraction methodologies, or proportional bias, as suggested by Bland–Altman plots (Supplementary Fig. 2).

The Bland–Altman plots (Supplementary Fig. 2) reveal proportional bias, with prediction errors increasing alongside the magnitude of predicted and actual values averages [46]. This trend suggests that our models tend to overestimate higher values while performing more accurately in predicting lower values across all three biomarkers. For HbA1c, this bias may be attributable to right-skewed distribution of the training data (skewness = 4.06), which includes a higher density of observations in the lower ranges and sparse representation in higher ranges (Supplementary Fig. 2). However, for SBP and DBP, where the skewness is less pronounced, this effect may reflect the inherent variability of these biomarkers.

The saliency maps show that our models focus on major retinal vessels for diabetes and hypertension classification, which may explain their limited sensitivity to the microvascular changes characteristic of these conditions (Supplementary Figs. 3–5). In contrast to the scattered regions of interest reported by Zhang et al. (2021) for diabetes detection [21], our findings emphasise the significance of the retinal vasculature, suggesting potential differences in model training mechanisms. Conversely, our models’ attention on vascular features and the optic disk for hypertension detection aligns with existing literature by Rim et al. (2020) [17] and Poplin et al. (2018) [45]. Future research could explore incorporating annotations of key signs to guide the models’ focus towards the relevant microvascular areas, potentially improving their sensitivity to the smaller blood vessels affected by diabetes and hypertension.

### Challenges in model performance and generalisability for personalised and preventive care

While the models showed promise in detecting the presence and control statuses (compared to non-diseased individuals) of diabetes and hypertension, they faced limitations in differentiating between poorly controlled and well-controlled cases, as well as in identifying pre-hypertension or pre-diabetes relative to healthy individuals. These challenges likely stem from the subtle retinal differences between these groups, making nuanced distinctions difficult, particularly when near clinical parametric thresholds. Moreover, the smaller sample sizes in the poorly controlled and well-controlled categories contributed to the widest 95% confidence interval observed, complicating reliable predictions. Refining these models is crucial to enhancing risk stratification and facilitating timely, targeted interventions.

Furthermore, the models exhibited varying degrees of generalisability across datasets. Stronger performance on the SP2 dataset was observed, likely due to its similarities with the SEED cohort in imaging protocol and demographics, as both are Singapore-based multiethnic population studies. In contrast, the BES dataset consistently achieved lower AUROC values, potentially due to differences in camera models and its exclusive focus on a Chinese population, which lacks the ethnic diversity present in the UKBB and SEED training datasets (Table 1). Notably, generalisation was more consistent for hypertension than for diabetes outcomes, suggesting that retinal biomarkers related for hypertension may be more stable across populations, whereas diabetes-related retinal changes exhibit greater variability.

### Limitations and outlook in the context of 3PM

Our study has several limitations. First, the exclusion of “ungradable” images through our quality assessment algorithm (Supplementary Material 1) was intended to minimise the risk of misclassification. However, this decision should be considered carefully when deploying these models in clinical settings, where lower-quality images may be encountered. Secondly, the ethnic imbalance between Asian and European cohorts in the training set may have influenced the performance of external test sets, which predominantly comprised Asian participants. Future research should aim for a more balanced cohort representation in external test sets to facilitate a fair assessment of the model’s generalisability.

Beyond these limitations, an important avenue for future research lies in enhancing the predictive utility of these models to encompass incidence prediction. While our models effectively classify the presence and control statuses of diabetes and hypertension, they do not yet forecast which individuals with pre-diabetes or pre-hypertension are at the highest risk of progression to disease onset. In this context, the potential integration of our models with emerging non-invasive biomarker profiling such as cell-free nucleic acids [47], metabolomics [48], and tear-based diagnostics [49–52] offers promising potential for multi-modal risk stratification. These technologies may provide molecular-level insights into individual disease trajectories and could potentially enhance the sensitivity and specificity of pre-disease detection. Advancing this predictive capability is crucial for strengthening the 3PM paradigm, enabling a shift from early detection to proactive, personalised prevention through tailored risk-based interventions.

## Conclusion and expert recommendations

This study demonstrates the potential of ViT-based deep learning models for retinal analysis in advancing the shift from reactive medicine to 3PM.

### Predictive approach

By enabling the stratification of control statuses and facilitating early detection of diabetes and hypertension, our models offer a unique and non-invasive alternative approach to conventional diagnostic methods, which often identify these conditions at later, more complicated stages. The ability to identify undiagnosed individuals, as well as those with poorly controlled conditions, supports earlier, risk-based interventions, aligning with a predictive approach to healthcare. More importantly, identifying individuals with pre-diabetes or pre-hypertension allows for timely interventions that can prevent disease progression, marking a significant advancement in predictive medicine.

### Targeted prevention in primary care

In primary care, where retinal imaging is increasingly available, these opportunistic screenings for diabetes and hypertension can facilitate timely interventions for those requiring urgent care, such as lifestyle changes or pharmacological treatments, that can halt or slow disease progression before it becomes clinically significant. This early intervention aligns with the principles of targeted prevention, ultimately improving patient outcomes and reducing the burden on healthcare systems.

### Personalisation of medical services

By stratifying the control statuses of diabetes and hypertension, our model supports personalised treatment decisions, ensuring that individuals with poorly controlled diabetes or hypertension receive timely treatment intensification while well-controlled patients avoid unnecessary further interventions. This tailored approach optimises disease management, enhances patient outcomes, and improves resource allocation within healthcare systems.

### Shaping the future of 3PM

To advance beyond the current state of the art, future research should focus on enhancing predictive capabilities, particularly in identifying individuals at risk of disease onset from pre-diabetes or pre-hypertension states. Integrating longitudinal data and refining personalised risk assessments will be critical in transitioning from early detection to true prevention, allowing for stratified, patient-specific interventions based on individual risk trajectories. These assessments may be potentially further enriched through non-invasive biomarker profiling from body fluids such as cell-free nucleic acids [47], metabolomics [48], and tears [49–52]. Additionally, improving model generalisability across diverse populations and enhancing robustness to real-world imaging variability are essential for clinical translation. By addressing these challenges, these models can potentially evolve into robust assistive tools for clinical decision-making, facilitating predictive, personalised, and preventative care within the 3PM framework.

## Supplementary Information

Below is the link to the electronic supplementary material.

Supplementary file1 (PPTX 3.31 MB)

Supplementary file2 (DOCX 40 KB)

## Data Availability

No datasets were generated or analysed during the current study.

## References

[1] Worldwide trends in hypertension prevalence and progress in treatment and control from 1990 to 2019: a pooled analysis of 1201 population-representative studies with 104 million participants. Lancet. 2021;398(10304):957–80. 10.1016/s0140-6736(21)01330-1. 10.1016/S0140-6736(21)01330-1 PMC8446938 34450083

[2] Ong KL, Stafford LK, Mclaughlin SA, Boyko EJ, Vollset SE, Smith AE, et al. Global, regional, and national burden of diabetes from 1990 to 2021, with projections of prevalence to 2050: a systematic analysis for the Global Burden of Disease Study 2021. Lancet. 2023;402(10397):203–34. 10.1016/s0140-6736(23)01301-6. 37356446 10.1016/S0140-6736(23)01301-6 PMC10364581

[3] Ogurtsova K, Guariguata L, Noël CB, Ruiz PL-D, Sacre JW, Karuranga S, et al. IDF diabetes Atlas: global estimates of undiagnosed diabetes in adults for 2021. Diab Res Clin Pract. 2022;2022(183):109118. 10.1016/j.diabres.2021.109118. 10.1016/j.diabres.2021.109118 34883189

[4] Kario K, Okura A, Hoshide S, Mogi M. The WHO Global report 2023 on hypertension warning the emerging hypertension burden in globe and its treatment strategy. Hypertens Res. vol 5. England: © 2024. The Author(s), under exclusive licence to The Japanese Society of Hypertension.; 2024. p. 1099–102. 10.1038/s41440-024-01622-w 38443614

[5] Man REK, Gan AHW, Fenwick EK, Gan ATL, Gupta P, Sabanayagam C, et al. Prevalence, determinants and association of unawareness of diabetes, hypertension and hypercholesterolemia with poor disease control in a multi-ethnic Asian population without cardiovascular disease. Popul Health Metr. 2019;17(1):17. 10.1186/s12963-019-0197-5. 31806040 10.1186/s12963-019-0197-5 PMC6896313

[6] Chow CK, Gupta R. Blood pressure control: a challenge to global health systems. Lancet. 2019;394(10199):613–5. 10.1016/s0140-6736(19)31293-0. 31327567 10.1016/S0140-6736(19)31293-0

[7] Golubnitschaja O, Baban B, Boniolo G, Wang W, Bubnov R, Kapalla M, et al. Medicine in the early twenty-first century: paradigm and anticipation - EPMA position paper 2016. EPMA J. 2016;7(1):23. 10.1186/s13167-016-0072-4. 27800037 10.1186/s13167-016-0072-4 PMC5078893

[8] Wang W, Yan Y, Guo Z, Hou H, Garcia M, Tan X, et al. All around suboptimal health - a joint position paper of the Suboptimal Health Study Consortium and European Association for Predictive. Preventive and Personalised Medicine EPMA J. 2021;12(4):403–33. 10.1007/s13167-021-00253-2. 34539937 10.1007/s13167-021-00253-2 PMC8435766

[9] Duarte AA, Mohsin S, Golubnitschaja O. Diabetes care in figures: current pitfalls and future scenario. EPMA J. 2018;9(2):125–31. 10.1007/s13167-018-0133-y. 29896313 10.1007/s13167-018-0133-y PMC5972141

[10] Golubnitschaja O, Kinkorova J, Costigliola V. Predictive, Preventive and personalised medicine as the hardcore of ‘Horizon 2020’: EPMA position paper. EPMA J. 2014;5(1):6. 10.1186/1878-5085-5-6. 24708704 10.1186/1878-5085-5-6 PMC3985551

[11] Zoungas S, Chalmers J, Ninomiya T, Li Q, Cooper ME, Colagiuri S, et al. Association of HbA1c levels with vascular complications and death in patients with type 2 diabetes: evidence of glycaemic thresholds. Diabetologia. 2012;55(3):636–43. 10.1007/s00125-011-2404-1. 22186981 10.1007/s00125-011-2404-1

[12] Zhou D, Xi B, Zhao M, Wang L, Veeranki SP. Uncontrolled hypertension increases risk of all-cause and cardiovascular disease mortality in US adults zthe NHANES III Linked Mortality Study. Sci Rep. 2018;8(1):9418. 10.1038/s41598-018-27377-2. 29925884 10.1038/s41598-018-27377-2 PMC6010458

[13] Davies MJ, Aroda VR, Collins BS, Gabbay RA, Green J, Maruthur NM, et al. Management of hyperglycemia in type 2 diabetes, 2022. A Consensus Report by the American Diabetes Association (ADA) and the European Association for the Study of Diabetes (EASD). Diab Care. 2022;45(11):2753–86. 10.2337/dci22-0034. 10.2337/dci22-0034 PMC10008140 36148880

[14] Melville S, Byrd JB. Personalized medicine and the treatment of hypertension. Curr Hypertens Rep. 2019;21(2):13. 10.1007/s11906-019-0921-3. 30747306 10.1007/s11906-019-0921-3 PMC6594382

[15] Sheng B, Pushpanathan K, Guan Z, Lim QH, Lim ZW, Yew SME, et al. Artificial intelligence for diabetes care: current and future prospects. Lancet Diabetes Endocrinol. 2024;12(8):569–95. 10.1016/s2213-8587(24)00154-2. 39054035 10.1016/S2213-8587(24)00154-2

[16] Wagner SK, Fu DJ, Faes L, Liu X, Huemer J, Khalid H, et al. Insights into systemic disease through retinal imaging-based oculomics. Transl Vis Sci Technol. 2020;9(2):6. 10.1167/tvst.9.2.6. 32704412 10.1167/tvst.9.2.6 PMC7343674

[17] Rim TH, Lee G, Kim Y, Tham Y-C, Lee CJ, Baik SJ, et al. Prediction of systemic biomarkers from retinal photographs: development and validation of deep-learning algorithms. Lancet Digit Health. 2020;2(10):e526–36. 10.1016/s2589-7500(20)30216-8. 33328047 10.1016/S2589-7500(20)30216-8

[18] Peng Q, Tseng R, Tham YC, Cheng CY, Rim TH. Detection of systemic diseases from ocular images using artificial intelligence: a systematic review. Asia Pac J Ophthalmol (Phila). 2022;11(2):126–39. 10.1097/apo.0000000000000515. 35533332 10.1097/APO.0000000000000515

[19] Tan YY, Kang HG, Lee CJ, Kim SS, Park S, Thakur S, et al. Prognostic potentials of AI in ophthalmology: systemic disease forecasting via retinal imaging. Eye Vis (Lond). 2024;11(1):17. 10.1186/s40662-024-00384-3. 38711111 10.1186/s40662-024-00384-3 PMC11071258

[20] Yun JS, Kim J, Jung SH, Cha SA, Ko SH, Ahn YB, et al. A deep learning model for screening type 2 diabetes from retinal photographs. Nutr Metab Cardiovasc Dis. 2022;32(5):1218–26. 10.1016/J.NUMECD.2022.01.010. 35197214 10.1016/j.numecd.2022.01.010 PMC9018521

[21] Zhang K, Liu X, Xu J, Yuan J, Cai W, Chen T, et al. Deep-learning models for the detection and incidence prediction of chronic kidney disease and type 2 diabetes from retinal fundus images. Nat Biomed Eng. 2021;5(6):533–45. 10.1038/S41551-021-00745-6. 34131321 10.1038/s41551-021-00745-6

[22] Zhang L, Yuan M, An Z, Zhao X, Wu H, Li H, et al. Prediction of hypertension, hyperglycemia and dyslipidemia from retinal fundus photographs via deep learning: a cross-sectional study of chronic diseases in central China. PLoS ONE. 2020;15(5):e0233166. 10.1371/JOURNAL.PONE.0233166. 32407418 10.1371/journal.pone.0233166 PMC7224473

[23] Ragab M, Al-Ghamdi ASAM, Fakieh B, Choudhry H, Mansour RF, Koundal D. Prediction of diabetes through retinal images using deep neural network. Comput Intel Neurosci. 2022;2022:7887908. 10.1155/2022/7887908. 10.1155/2022/7887908 PMC9187442 35694596

[24] Al-Absi HRH, Pai A, Naeem U, Mohamed FK, Arya S, Sbeit RA, et al. DiaNet v2 deep learning based method for diabetes diagnosis using retinal images. Sci Rep. 2024;14(1):1595. 10.1038/s41598-023-49677-y. 38238377 10.1038/s41598-023-49677-y PMC10796402

[25] Dai G, He W, Xu L, Pazo EE, Lin T, Liu S, et al. Exploring the effect of hypertension on retinal microvasculature using deep learning on East Asian population. PloS ONE. 2020;15(3):e0230111. 10.1371/JOURNAL.PONE.0230111. 32134976 10.1371/journal.pone.0230111 PMC7058325

[26] Baharoon M, Almatar H, Alduhayan R, Aldebasi T, Alahmadi BO, Bokhari Y, et al. HyMNet: a multimodal deep learning system for hypertension classification using fundus photographs and cardiometabolic risk factors. ArXiv. 2023; abs/2310.01099. 10.3390/bioengineering11111080 PMC11591283 39593740

[27] Raghu M, Unterthiner T, Kornblith S, Zhang C, Dosovitskiy A. Do vision transformers see like convolutional neural networks? Adv Neural Inform Proc Syst. 2021;34:12116–28.

[28] Goh JHL, Ang E, Srinivasan S, Lei X, Loh J, Quek TC, et al. Comparative analysis of vision transformers and conventional convolutional neural networks in detecting referable diabetic retinopathy. Ophthalmol Sci. 2024;4(6):100552. 10.1016/j.xops.2024.100552. 39165694 10.1016/j.xops.2024.100552 PMC11334703

[29] Bertele N, Karabatsiakis A, Buss C, Talmon A. How biomarker patterns can be utilized to identify individuals with a high disease burden: a bioinformatics approach towards predictive, preventive, and personalized (3P) medicine. EPMA J. 2021;12(4):507–16. 10.1007/s13167-021-00255-0. 34950251 10.1007/s13167-021-00255-0 PMC8648886

[30] Smokovski I, Steinle N, Behnke A, Bhaskar SMM, Grech G, Richter K, et al. Digital biomarkers: 3PM approach revolutionizing chronic disease management - EPMA 2024 position. EPMA J. 2024;15(2):149–62. 10.1007/s13167-024-00364-6. 38841615 10.1007/s13167-024-00364-6 PMC11147994

[31] Sudlow C, Gallacher J, Allen N, Beral V, Burton P, Danesh J, et al. UK biobank: an open access resource for identifying the causes of a wide range of complex diseases of middle and old age. PLoS Med. 2015;12(3):e1001779. 10.1371/journal.pmed.1001779. 25826379 10.1371/journal.pmed.1001779 PMC4380465

[32] Majithia S, Tham YC, Chee ML, Nusinovici S, Teo CL, Thakur S, et al. Cohort profile: the Singapore Epidemiology of Eye Diseases study (SEED). Int J Epidemiol. 2021;50(1):41–52. 10.1093/ije/dyaa238. 33393587 10.1093/ije/dyaa238

[33] Hughes K, Yeo PP, Lun KC, Thai AC, Sothy SP, Wang KW, et al. Cardiovascular diseases in Chinese, Malays, and Indians in Singapore. II. Differences in risk factor levels. J Epidemiol Commun Health. 1990;44(1):29–35. 10.1136/jech.44.1.29. 10.1136/jech.44.1.29 PMC1060593 2348145

[34] Jonas JB, Xu L, Wang YX. The Beijing eye study. Acta Ophthalmol. 2009;87(3):247–61. 10.1111/j.1755-3768.2008.01385.x. 19426355 10.1111/j.1755-3768.2008.01385.x

[35] Huang C, Jiang Y, Yang X, Wei C, Chen H, Xiong W, et al. Enhancing retinal fundus image quality assessment with Swin-Transformer–based learning across multiple color-spaces. Transl Vis Sci Technol. 2024;13(4):8. 10.1167/tvst.13.4.8. 38568606 10.1167/tvst.13.4.8 PMC10996994

[36] Priyadharsini C. Retinal image enhancement based on color dominance of image. Sci Rep. 2023;13:7172. 10.1038/s41598-023-34212-w. 37138000 10.1038/s41598-023-34212-w PMC10156681

[37] Elsayed NA, Aleppo G, Bannuru RR, Bruemmer D, Collins BS, Ekhlaspour L, et al. 6 lycemic goals and hypoglycemia: standards of care in diabetes. Diab Care. 2024;47(Supplement_1):S111–25. 10.2337/dc24-s006. 10.2337/dc24-S006 PMC10725808 38078586

[38] Whelton PK, Carey RM, Aronow WS, Casey DE, Collins KJ, Dennison Himmelfarb C, et al. 2017 ACC/AHA/AAPA/ABC/ACPM/AGS/APhA/ASH/ASPC/NMA/PCNA guideline for the prevention, detection, evaluation, and management of high blood pressure in adults: a report of the American College of Cardiology/American Heart Association Task Force on Clinical Pr. Hypertension. 2018;71(6):e13–115. 10.1161/hyp.0000000000000065. 29133356 10.1161/HYP.0000000000000065

[39] Dosovitskiy A, Beyer L, Kolesnikov A, Weissenborn D, Zhai X, Unterthiner T, et al. An image is worth 16x16 words: transformers for image recognition at scale. ArXiv. 2020 abs/2010.11929.

[40] Wahid JA, Mingliang X, Ayoub M, Husssain S, Li L, Shi L. A hybrid ResNet-ViT approach to bridge the global and local features for myocardial infarction detection. Sci Rep. 2024;14(1):4359. 10.1038/s41598-024-54846-8. 38388668 10.1038/s41598-024-54846-8 PMC10883929

[41] Selvaraju RR, Cogswell M, Das A, Vedantam R, Parikh D, Batra D. Grad-CAM: visual explanations from deep networks via gradient-based localization. Int J Comput Vision. 2020;128(2):336–59. 10.1007/s11263-019-01228-7.

[42] Ferrari P. the National Coordinators for the Reasons for not Intensifying Antihypertensive Treatment. Reasons for therapeutic inertia when managing hypertension in clinical practice in non-Western countries. J Human Hyper. 2009;23(3):151–9. 10.1038/jhh.2008.117. 10.1038/jhh.2008.117 18784735

[43] Andreozzi F, Candido R, Corrao S, Fornengo R, Giancaterini A, Ponzani P, et al. Clinical inertia is the enemy of therapeutic success in the management of diabetes and its complications: a narrative literature review. Diabetol Metabol Synd. 2020;12(1):1–1. 10.1186/s13098-020-00559-7. 10.1186/s13098-020-00559-7 PMC7301473 32565924

[44] Dungan KM, Braithwaite SS, Preiser JC. Stress hyperglycaemia. Lancet. 2009;373(9677):1798–807. 10.1016/s0140-6736(09)60553-5. 19465235 10.1016/S0140-6736(09)60553-5 PMC3144755

[45] Poplin R, Varadarajan AV, Blumer K, Liu Y, Mcconnell MV, Corrado GS, et al. Prediction of cardiovascular risk factors from retinal fundus photographs via deep learning. Nat Biomed Eng. 2018;2(3):158–64. 10.1038/s41551-018-0195-0. 31015713 10.1038/s41551-018-0195-0

[46] Giavarina D. Understanding Bland Altman analysis. Biochem Med (Zagreb). 2015;25(2):141–51. 10.11613/bm.2015.015. 26110027 10.11613/BM.2015.015 PMC4470095

[47] Crigna AT, Samec M, Koklesova L, Liskova A, Giordano FA, Kubatka P, et al. Cell-free nucleic acid patterns in disease prediction and monitoring-hype or hope? EPMA J. 2020;11(4):603–27. 10.1007/s13167-020-00226-x. 33144898 10.1007/s13167-020-00226-x PMC7594983

[48] Zhang Q, Wang N, Rui Y, Xia Y, Xiong S, Xia X. New insight of metabolomics in ocular diseases in the context of 3P medicine. EPMA J. 2023;14(1):53–71. 10.1007/s13167-023-00313-9. 36866159 10.1007/s13167-023-00313-9 PMC9971428

[49] Brunmair J, Bileck A, Schmidl D, Hagn G, Meier-Menches SM, Hommer N, et al. Metabolic phenotyping of tear fluid as a prognostic tool for personalised medicine exemplified by T2DM patients. EPMA J. 2022;13(1):107–23. 10.1007/s13167-022-00272-7. 35265228 10.1007/s13167-022-00272-7 PMC8897537

[50] Zhan X, Li J, Guo Y, Golubnitschaja O. Mass spectrometry analysis of human tear fluid biomarkers specific for ocular and systemic diseases in the context of 3P medicine. EPMA J. 2021;12(4):449–75. 10.1007/s13167-021-00265-y. 34876936 10.1007/s13167-021-00265-y PMC8639411

[51] Kropp M, De Clerck E, Vo TKS, Thumann G, Costigliola V, Golubnitschaja O. Short communication: unique metabolic signature of proliferative retinopathy in the tear fluid of diabetic patients with comorbidities - preliminary data for PPPM validation. EPMA J. 2023;14(1):43–51. 10.1007/s13167-023-00318-4. 36845280 10.1007/s13167-023-00318-4 PMC9944425

[52] Golubnitschaja O, Polivka J Jr, Potuznik P, Pesta M, Stetkarova I, Mazurakova A, et al. The paradigm change from reactive medical services to 3PM in ischemic stroke: a holistic approach utilising tear fluid multi-omics, mitochondria as a vital biosensor and AI-based multi-professional data interpretation. EPMA J. 2024;15(1):1–23. 10.1007/s13167-024-00356-6. 38463624 10.1007/s13167-024-00356-6 PMC10923756

