# Supplementary material for: Vision transformer-based stratification of pre/diabetic and pre/hypertensive patients from retinal photographs for 3PM applications
Source: EPMA J. 2025 May 20;16(2):519–33. doi: 10.1007/s13167-025-00412-9 (PMC12106178; doi:10.1007/s13167-025-00412-9)
Supplement: Supplementary file 1 — Supplementary file1 (PPTX 3.31 MB) [file 13167_2025_412_MOESM1_ESM.pptx]

## Slide 1
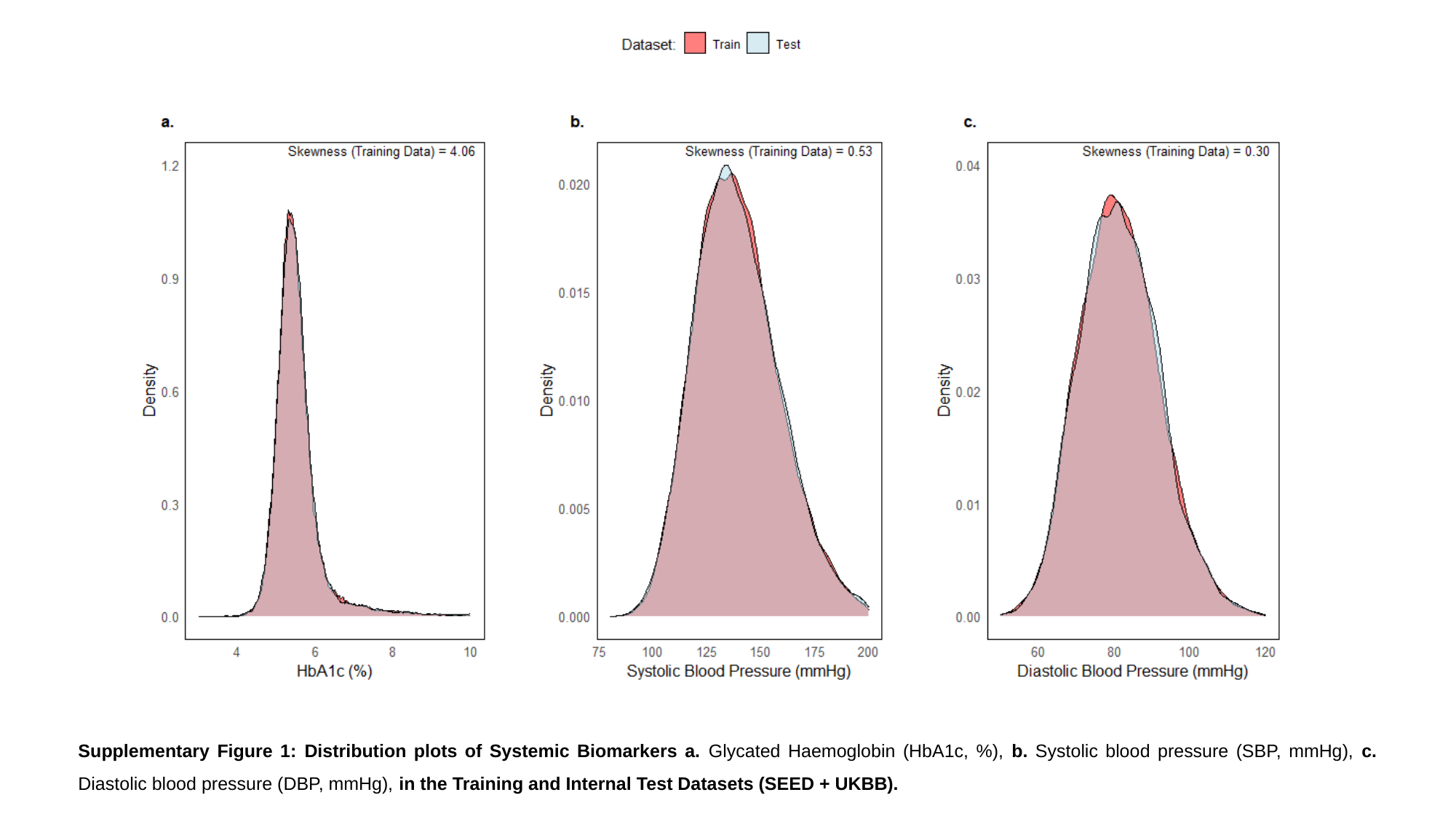

Supplementary Figure 1: Distribution plots of Systemic Biomarkers a. Glycated Haemoglobin (HbA1c, %), b. Systolic blood pressure (SBP, mmHg), c. Diastolic blood pressure (DBP, mmHg), in the Training and Internal Test Datasets (SEED + UKBB).

## Slide 2
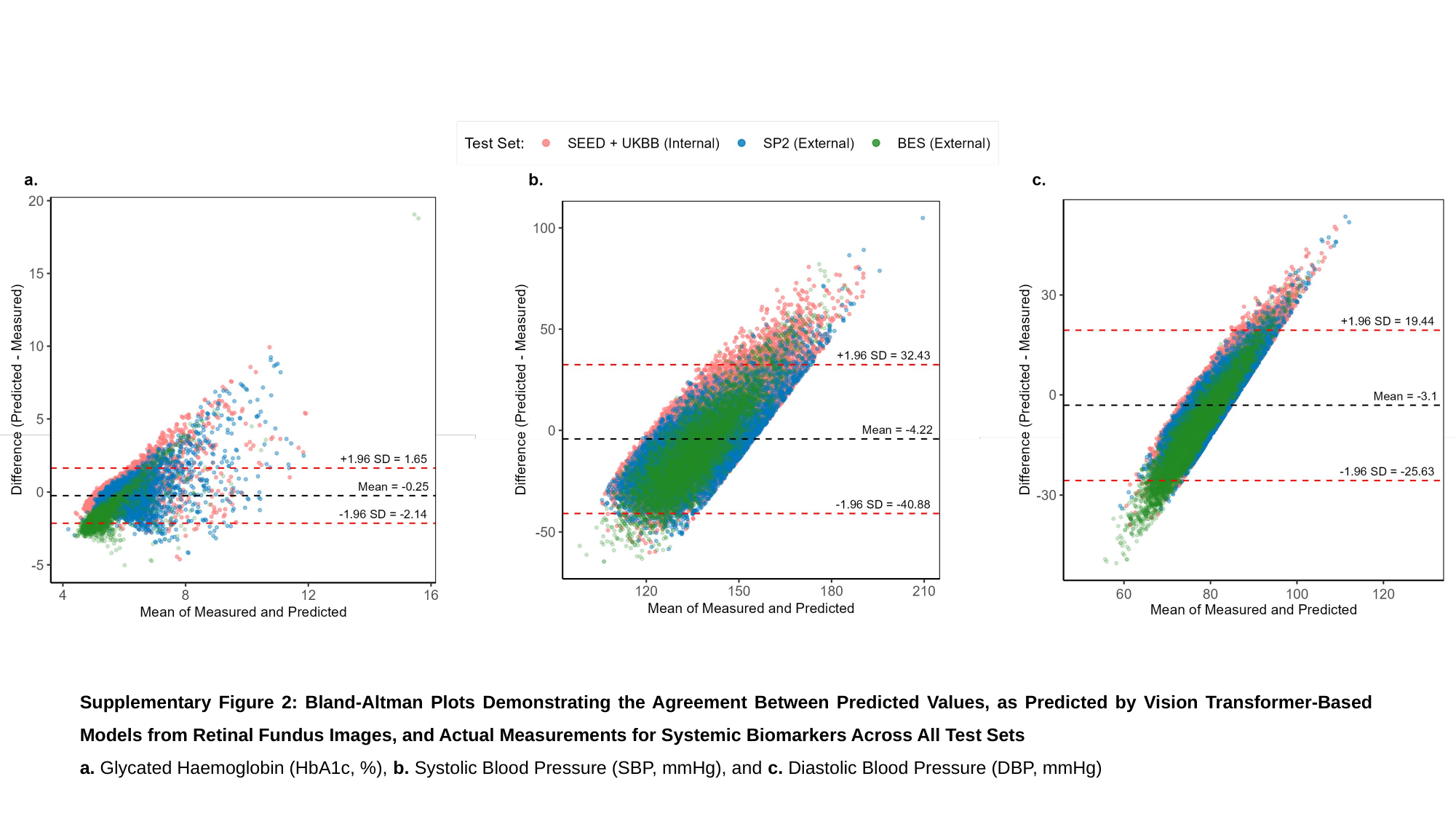

Supplementary Figure 2: Bland-Altman Plots Demonstrating the Agreement Between Predicted Values, as Predicted by Vision Transformer-Based Models from Retinal Fundus Images, and Actual Measurements for Systemic Biomarkers Across All Test Sets
a. Glycated Haemoglobin (HbA1c, %), b. Systolic Blood Pressure (SBP, mmHg), and c. Diastolic Blood Pressure (DBP, mmHg)

## Slide 3
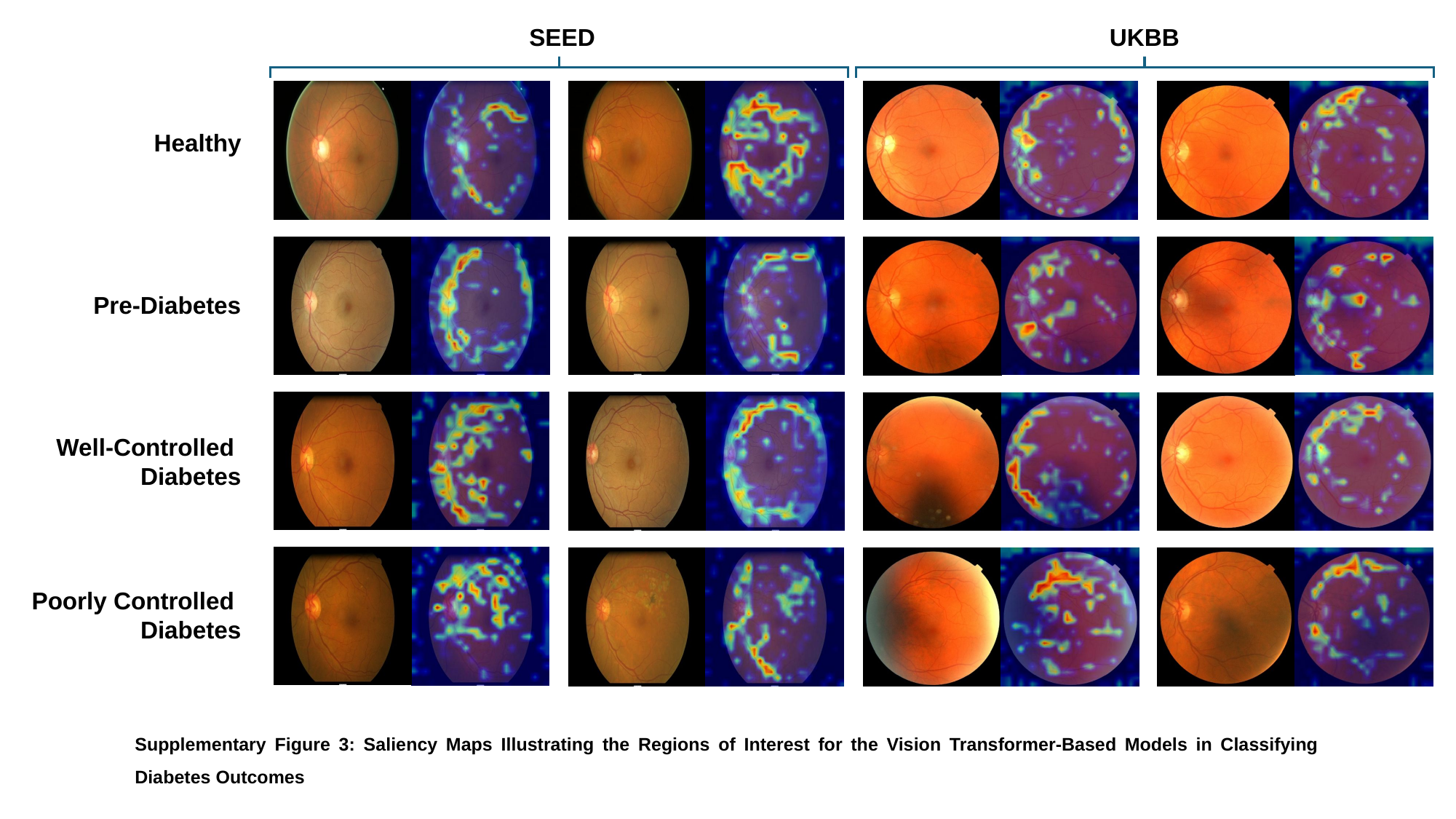

SEED
UKBB
Pre-Diabetes
Well-Controlled
Diabetes
Poorly Controlled
Diabetes
Supplementary Figure 3: Saliency Maps Illustrating the Regions of Interest for the Vision Transformer-Based Models in Classifying Diabetes Outcomes
Healthy

## Slide 4
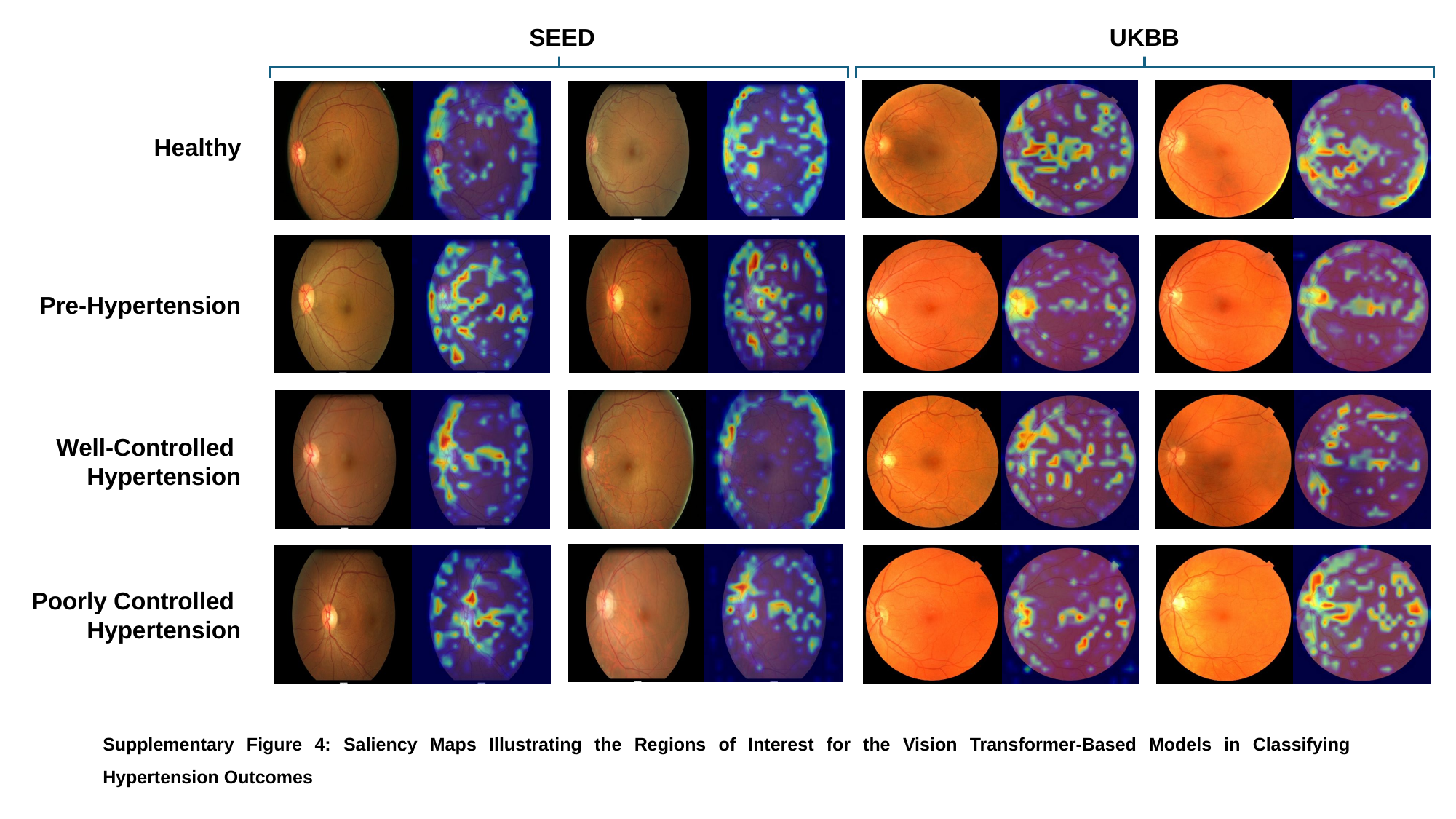

SEED
UKBB
Healthy
Pre-Hypertension
Well-Controlled
Hypertension
Poorly Controlled
Hypertension
Supplementary Figure 4: Saliency Maps Illustrating the Regions of Interest for the Vision Transformer-Based Models in Classifying Hypertension Outcomes

## Slide 5
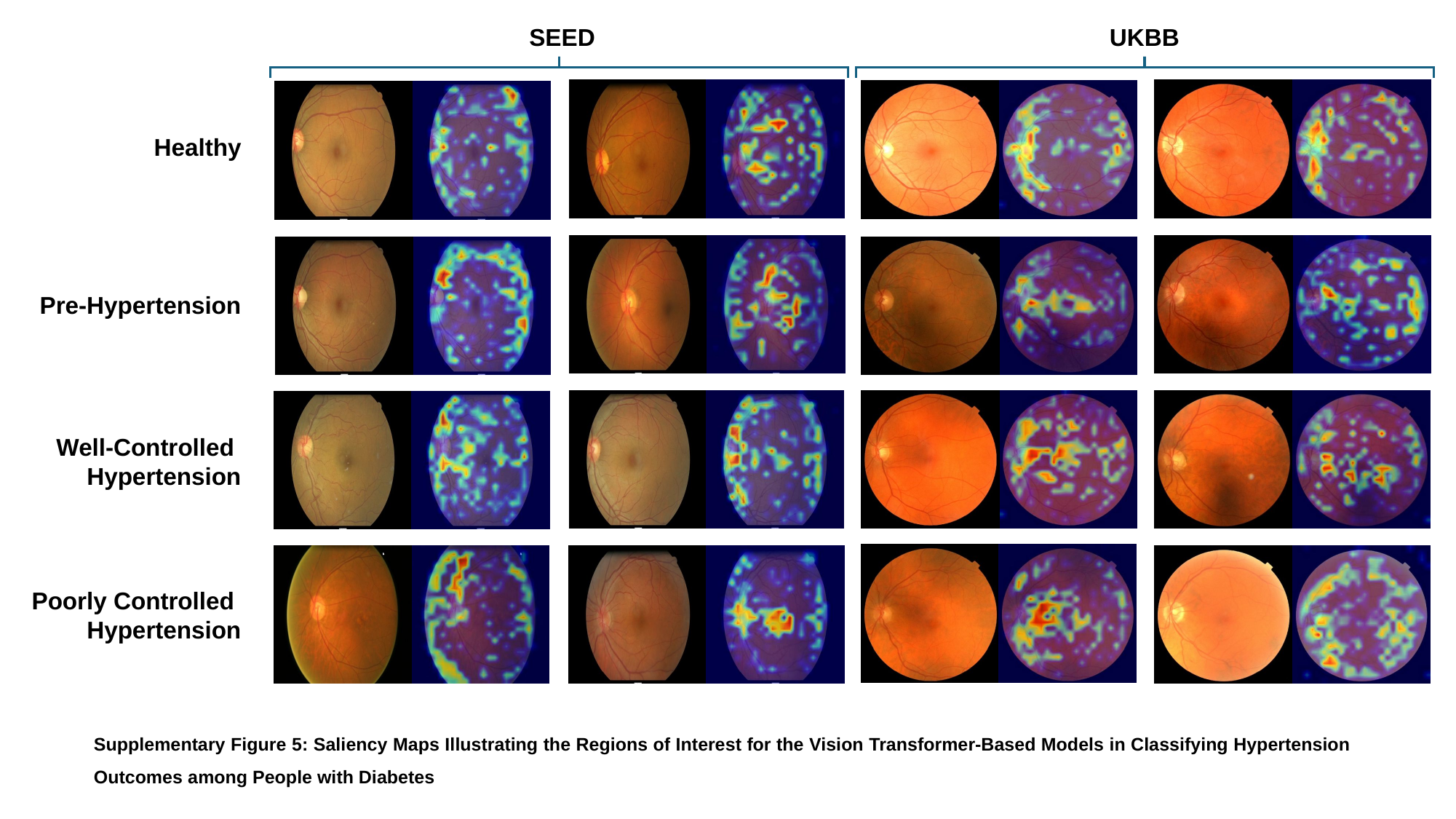

SEED
UKBB
Supplementary Figure 5: Saliency Maps Illustrating the Regions of Interest for the Vision Transformer-Based Models in Classifying Hypertension Outcomes among People with Diabetes
Healthy
Pre-Hypertension
Well-Controlled
Hypertension
Poorly Controlled
Hypertension
