# Supplementary material for: Vision transformer-based stratification of pre/diabetic and pre/hypertensive patients from retinal photographs for 3PM applications
Source: EPMA J. 2025 May 20;16(2):519–33. doi: 10.1007/s13167-025-00412-9 (PMC12106178; doi:10.1007/s13167-025-00412-9)
Supplement: Supplementary file 2 — Supplementary file2 (DOCX 40 KB) [file 13167_2025_412_MOESM2_ESM.docx]

## **Supplementary Material 1: Details on Image Pre-Processing**

The in-house deep learning model was developed using the Swin Transformer V2 architecture and trained on 46,969 images sourced from multiple datasets, including the UKBB, SEED, EyeQ/EyePACS, and a local clinical dataset. The model achieved an AUC of 0.99 on the internal test set and 0.93 on the external BES test set (6,663 images). Pre-processing involved resizing images to 256 x 256 pixels and normalising them, with data augmentation techniques including random horizontal flipping, scaling, and rotation. Initial weights are loaded from pre-trained models on ImageNet.

The model then assigns a score to each image, categorising them as either "gradable," indicating clear visualisation of all fundus components, or "ungradable," denoting significant issues that may hinder accurate diagnosis, even by trained graders. This classification relies on whether the image scores exceed a predefined threshold. This algorithm was applied to all four datasets in our study to filter out poor-quality images. When multiple images of the same eye were available, the algorithm identified the highest-quality image based on the lowest score.

## **Supplementary Table 1: Classification Criteria for Diabetes and Hypertension Status and Control Levels**

| **Disease Status** | **Definition*** |
| --- | --- |
| Pre-Diabetes | **All** of the following must be satisfied:   - 5.6 $\leq$Fasting plasma glucose $<$ 7.0 mmol/L *(only applicable for BES and SP2)* **or** 5.7% $\leq$ HbA1c $<$ 6.5% - No self-reported history of diabetes - No use of diabetic medication |
| Diabetes | **At least one** of the following must be satisfied:   - Random plasma glucose $\geq$11.1 mmol/L *(only applicable for SEED)* - Fasting plasma glucose $\geq$7.0 mmol/L *(only applicable for BES and SP2)* - HbA1c $\geq$ 6.5% - Physician diagnosis of diabetes *(only applicable for UKBB)* - Self-reported history of diabetes - Use of diabetic medication |
| Well-Controlled Diabetes | **All** of the following must be satisfied:   - HbA1c $<$7.0% - Use of diabetic medication |
| Poorly-Controlled Diabetes | **All** of the following must be satisfied:   - HbA1c $\geq$7.0% - Use of diabetic medication |
| Pre-Hypertension | **All** of the following must be satisfied:   - 120 $\leq$SBP $<$140 mmHg **or** 80 $\leq$DBP $<$90 mmHg - No self-reported history of hypertension - No use of hypertensive medication |
| Hypertension | **At least one** of the following must be satisfied:   - SBP $\geq$140 mmHg - DBP $\geq$90 mmHg - Self-reported history of hypertension *(not applicable for UKBB)* - Use of hypertensive medication |
| Well-Controlled Hypertension | **All** of the following must be satisfied:   - SBP $<$140 mmHg - DBP $<$ 90 mmHg - Use of hypertensive medication |
| Poorly-Controlled Hypertension | **All** of the following must be satisfied:   - SBP $\geq$140 mmHg **or** DBP $\geq$90 mmHg - Use of hypertensive medication |

*Definitions were aligned with guidelines from the American Diabetes Association and the American Heart Association

## **Supplementary Table 2: Number of Individuals and Retinal Images Used for Training, Validation, and Testing of Each Model for Different Diabetes Use Cases**

| Model / Use Case | Training Set  (SEED + UKBB) | Validation Set  (SEED + UKBB) | Test Set | | |
| --- | --- | --- | --- | --- | --- |
|  |  |  | **SEED + UKBB (Internal)** | **SP2 (External)** | **BES (External)** |
| Presence of Diabetes | | | | | |
| Number of Images | 92,003 | 19,744 | 19,747 | 7793 | 3415 |
| Number of Individuals | 49,706 | 10,650 | 10,654 | 3999 | 1771 |
| Poorly Controlled Diabetes (Compared to Non-Diseased Individuals) | | | | | |
| Number of Images | Fine-tuned on Yes/No model | | 18,640 | 6818 | 2539 |
| Number of Individuals |  |  | 10,049 | 3493 | 1315 |
| Well-Controlled Diabetes (Compared to Non-Diseased Individuals) | | | | | |
| Number of Images | Fine-tuned on Yes/No model | | 18,537 | 6587 | 2862 |
| Number of Individuals |  |  | 9991 | 3373 | 1483 |
| Poorly Controlled Diabetes vs. Well-Controlled Diabetes | | | | | |
| Number of Images | Fine-tuned on Yes/No model | | 967 | 421 | 457 |
| Number of Individuals |  |  | 542 | 224 | 238 |
| Pre-Diabetes (Compared to Healthy Individuals) | | | | | |
| Number of Images | 84,350 | 18,046 | 18,124 | 6492 | 2472 |
| Number of Individuals | 45,488 | 9747 | 9,750 | 3321 | 1280 |

The SEED + UKBB data was allocated with a 70% training, 15% validation, and 15% testing split by individual

## **Supplementary Table 3: Number of Individuals and Retinal Images Used for Training, Validation, and Testing of Each Model for Different Hypertension Use Cases**

| Model / Use Case | Training Set  (SEED + UKBB) | Validation Set  (SEED + UKBB) | Test Sets | | |
| --- | --- | --- | --- | --- | --- |
|  |  |  | **SEED + UKBB (Internal)** | **SP2 (External)** | **BES (External)** |
| Presence of Hypertension | | | | | |
| Number of Images | 91,034 | 19,515 | 7632 | 7705 | 5969 |
| Number of Individuals | 49,179 | 10,538 | 3917 | 3955 | 3097 |
| Poorly Controlled Hypertension (Compared to Non-Diseased Individuals) | | | | | |
| Number of Images | Fine-tuned on Yes/No model | | 11,330 | 5415 | 3386 |
| Number of Individuals |  |  | 6102 | 2772 | 1745 |
| Well-Controlled Hypertension (Compared to Non-Diseased Individuals) | | | | | |
| Number of Images | Fine-tuned on Yes/No model | | 9916 | 4965 | 3948 |
| Number of Individuals |  |  | 5331 | 2524 | 2039 |
| Poorly-Controlled Hypertension vs. Well-Controlled Hypertension | | | | | |
| Number of Images | Fine-tuned on Yes/No model | | 4280 | 1254 | 2490 |
| Number of Individuals |  |  | 2345 | 664 | 1300 |
| Pre-Hypertension (Compared to Healthy Individuals) | | | | | |
| Number of Images | 39,669 | 8454 | 4551 | 4563 | 2422 |
| Number of Individuals | 21,202 | 4543 | 2310 | 2316 | 1242 |

The SEED + UKBB data was allocated with a 70% training, 15% validation, and 15% testing split by individual

## **Supplementary Table 4: Regression Analysis Results for Predicting Biomarkers Associated with Diabetes and Hypertension**

| **Test Set** | **Root Mean Squared Error (RMSE)** | **Mean Absolute Error (MAE)** | **Standard Deviation (SD)** | **Coefficient of Determination (R^2^)** | **Systematic Bias (p-value of t-test)** | **Proportional Bias (p-value of regression slope test)** |
| --- | --- | --- | --- | --- | --- | --- |
| **Glycated Haemoglobin Serum Concentration (HbA1c, %)** | | | | | | |
| SEED + UKBB (Internal) | 0.656 | 0.386 | 0.656 | 0.264 | <0.001 | <0.001 |
| SP2 (External) | 1.042 | 0.670 | 1.029 | 0.168 | <0.001 | <0.001 |
| BES (External) | 2.008 | 1.856 | 1.142 | 0.067 | <0.001 | <0.001 |
| **Systolic Blood Pressure (SBP, mmHg)** | | | | | | |
| SEED + UKBB (Internal) | 17.855 | 14.119 | 17.852 | 0.191 | <0.001 | <0.001 |
| SP2 (External) | 19.587 | 16.074 | 17.282 | 0.294 | <0.001 | <0.001 |
| BES (External) | 22.506 | 18.533 | 19.905 | 0.071 | <0.001 | <0.001 |
| **Diastolic Blood Pressure (DBP, mmHg)** | | | | | | |
| SEED + UKBB (Internal) | 10.198 | 8.092 | 10.197 | 0.086 | 0.162 | <0.001 |
| SP2 (External) | 10.827 | 8.884 | 10.087 | 0.126 | <0.001 | <0.001 |
| BES (External) | 17.230 | 14.591 | 12.519 | 0.019 | <0.001 | <0.001 |
